# Death of Sir Everard Home

**Published:** 1832-10

**Authors:** 


### OBITUARY.

### DEATH OF SIR EVERARD HOME.

Sir Everard Home died, at Chelsea, on Friday, the 31st ultimo, in the seventy-seventh year of his age. Sir Everard was of Scotch extraction, and his connexion with the Hunters brought him into notice at an early period. At one time he enjoyed high consideration, and the more substantial advantage of an extensive practice. He had the good fortune to be appointed one of the surgeons to St. George's Hospital, which, added to various works which attained a considerable degree of celebrity, contributed to extend his reputation, the advantages of which he lived nearly half a century to enjoy. Sir Everard Home was the author of "Practical Observations on Stricture," of "Lectures on Comparative Anatomy," and of various papers in the "Philosophical Transactions." In 1813 he was created a baronet by his late Majesty; he was also sergeant-surgeon to the King, and surgeon to Chelsea Hospital. For several years before his death, Sir Everard had retired from public life, and we believe that his habits, together with his advanced years, had led to considerable bodily infirmity.

